# Editorial: Targeted Antigen Delivery: Bridging Innate and Adaptive Immunity

**DOI:** 10.3389/fimmu.2019.00368

**Published:** 2019-03-05

**Authors:** Bénédicte Manoury, Piergiuseppe De Berardinis

**Affiliations:** ^1^Institut Necker Enfants Malades, INSERM U1151-CNRS UMR8253, Paris, France; ^2^Institute of Protein Biochemistry, CNR, Naples, Italy

**Keywords:** targeted delivery, dendritic cells, innate and adaptive immunity, pattern recognition receptors (PRR), immunotherapy

*Those who are skilled can hit the target with one arrow. Whether one hits or not is entirely a matter of the two words “talent” and “learning”*.

*Harmony garden Yuan Mei (1716-1798)*

The aim of this topic is to provide an exhaustive as possible overview of currently used or developing targeting strategies for antigen delivery and of emerging approaches for simultaneous delivery of immunogenic molecules that can activate innate and adaptive responses.

To optimize T cell responses, immunologists focused on targeting antigenic proteins to professional antigen presenting cells of the immune system, like Dendritic Cells (DC), which also regulate innate immune responses, expressing various pattern recognition receptors (PRR), like Toll-like receptors, NOD-like receptors and cytosolic DNA and/or RNA sensors. Thus, developing strategies aim to exploit specialized uptake receptors constructing immunogens able to combine PRR ligands to antigen delivery via specific targeting.

This topic is a collection of 3 review, 5 mini-reviews, and 6 research articles which we hope will interest the readers and provide useful information for researchers more directly working on this field.

For their capability to be a bridge between innate and adaptive immunity, targeting of DC is an attractive approach to generate strong protective cellular responses against infectious diseases and cancer.

In order to optimize DC-based vaccine, Antonialli et al. reported the targeting of different DC subsets by using hybrids mAbs able to deliver antigens of interest to DC surface receptors. They found that using anti-DEC-205 or anti-DCIR mAbs in the presence of CPG ODN or bacterial flagellin, when the antigen was targeted to CD8^+^ DC subset, a specific proliferation of CD4^+^ T cells was induced able to produce pro-inflammatory cytokines, while targeting CD8^−^ DC with the same hybrid mAbs promoted specific antibody responses but no detectable pro-inflammatory CD4^+^ T cell response.

On a similar path line of DCs targeting, another research article of this issue by Gomes-Neto et al. described a vaccine formulation based on the use of filamentous bacteriophage fd carrying *Trypanosoma cruzi* (*T. cruzi*) antigenic determinants and its efficacy in the absence of exogenous adjuvant administration. Gomes-Neto et al. reported the ability of fd nanoparticles, which were previously demonstrated to be taken up by dendritic cells, to protect against mortality induced by a high inoculum dose of parasite in a mice model of *T. cruzi* infection.

Moreover, the research article by Sartorius et al. showed a further exploitation of bacteriophage fd nanoparticles to deliver immunologically active lipids together with antigenic peptides. It was demonstrated that the delivery of alphaGalactosylCeramide (αGalCer) like bacteriophage fd/αGalCer conjugates was able to repeatedly stimulate iNKT cells *in vitro* and *in vivo*, without inducing anergy. In addition, the authors found that co-delivery of αGalCer and CD8^+^ T cell antigens to APCs via bacteriophages strongly boosted the adaptive CD8^+^T cell response, and therapeutic vaccination with these phage conjugates was able to protect mice against subcutaneous tumor engraft.

Targeting of macrophages was also addressed in this topic. In particular, in an original research article van Dinther et al. proposed a strategy to target CD169+ macrophages, which are located in the marginal zone of the spleen and the sub capsular sinus in the lymph nodes. In previous work, the authors demonstrated that antigen delivery to CD169^+^ macrophages resulted in antigen presentation by DCs and activation of strong CD8^+^ T cell responses in mice. Here by targeting tumor antigenic proteins or peptides as anti-CD169 antibody-antigen conjugates, they showed induction of strong primary, memory, and recall response. Moreover, a protective immunity against melanoma was generated in mice injected with B16 melanoma cells.

Several review articles are also an important part of this topic providing a compendium of the current and envisaged strategy of antigen delivery and, importantly, an updated dissection of the basic mechanisms, which orchestrate the innate and adaptive response to antigen delivery.

The central role of dendritic cells in the “cross hair for the generation of tailored vaccines” was reviewed in this research topic issue by Gornati et al. Crucial key points as the adjuvant selection, the vehicle design and the choice of membrane receptor molecules to target were discussed in this article. A more precise DCs classification in order to dissect an accurate view of the DCs role and cues for more specific targeting was described. Overall, the complexity of the immune response to cancer was also addressed in order to envisage efficacious personalized therapies.

Since decades, amelioration of DC immunotherapy to fight cancer has been the focus of intensive work. The group of E Lion wrote an extensive review on strategies to increase anti-tumor immunity by interfering with the PD1/PDL-1 pathway such as the used of humanized antibodies, nanobodies, soluble PD1 and RNA interference. On the same line, an original research article by Takeda et al. described the role of a short form of Mycoplasma fermentas-derived diacylated lipoprotein (MALP2) (a TLR2/TLR6 ligand) in inducing tumor rejection when used with anti PDL-1 antibodies therapy. MALP2 was shown to induce tumor DC activation and CTL proliferation.

Also in this topic, a comprehensive review on how to manipulate dendritic cells for effective anti-tumor response in hematological malignancies was written by Cornel et al. This review focused on strategies to potentiate DCs, which can be generated in sufficient quantities from CD34^+^ hematopoietic and stem cells progenitors, to express tumor associated antigens, to mature, to polarize, to migrate, to cross-present antigens, aiming at improving their potency in anti-tumor response.

In another mini review, Guo et al. described recent advancement in the development of neo-antigen-based cancer vaccines, reporting current strategies for lymphoid organ targeting and on-going efforts in using direct injection of *ex vivo* pulsed dendritic cells vaccines. Moreover, these authors reviewed the use of biomaterials designed for passive delivery via antigen capture *in vivo*. In addition, the authors described the use of bulk nanomaterial made from silica microrod for enhancing cancer vaccines through constructing artificial antigen-presenting niche.

Recently, nanovaccines engineered to express antigens and adjuvants on the same nanocarrier can also be used instead of traditional vaccines (antigen and adjuvant are delivered separately). Bros et al. discussed the biochemical properties and the nature of these nanocarriers, which can affect the uptake and trafficking of nanovaccines, in a mini-review.

Adjuvants containing nucleic acid (NA) targeting TLR9 have been shown to boost anti-cancer immune responses. Nucleic acids can also be recognized by cytosolic receptors and trigger cGAS-STING/RIG1-MAVS pathways to produce proinflammatory cytokines. The review of Iurescia et al. described how these NA have been used in recent clinical trials and represented a novel strategy for cancer immunotherapy.

On the other hand, Anchim et al. provide an original research article investigating the role of epitope display on Adenovirus capside. paving the way for the development of Ad vaccines able to trigger specific response against the epitope inserted and not against the transgene.

Another mini review article by Hos et al. focused on “approaches to improve chemically defined synthetic peptide vaccines”. The history of peptides vaccines, made of beneficial outcomes in preclinical models and mixed results obtained in clinical trials is summarized. An aim of this mini review was to report the novel options for a rationale design of peptide vaccines. Moreover, strategies for biochemical visualization and tracking of peptide vaccines at the molecular and subcellular level were described and discussed.

The nature and biology of the targeted cells emphasizes the importance of investigating the molecular mechanisms that modulate the successful delivery of immunogenic molecules. Thus, in this topic, Aksoy et al. summarized the role of the different isoforms of phosphoinositide-3 kinases (PI3Ks) proteins generating phosphoinositides lipids in DC biology, focusing in antigen presentation and PRR stimulation.

In conclusion, this research topic provides a general frame of the most efficient way to mount a sustained immune response by targeting antigen to antigen presenting cells, and a description of the exploitable strategies to trigger both innate and adaptive efficient immune responses.

## Author Contributions

All authors listed have made a substantial, direct and intellectual contribution to the work, and approved it for publication.

### Conflict of Interest Statement

PD is inventor of the patent n PCT/IB2018/050525 WO2018/138696 entitled “Phages conjugates and uses thereof.” The remaining author declares that the research was conducted in the absence of any commercial or financial relationships that could be construed as a potential conflict of interest.

